# Accounting for Genotype-by-Environment Interactions and Residual Genetic Variation in Genomic Selection for Water-Soluble Carbohydrate Concentration in Wheat

**DOI:** 10.1534/g3.118.200038

**Published:** 2018-04-16

**Authors:** Ben Ovenden, Andrew Milgate, Len J. Wade, Greg J. Rebetzke, James B. Holland

**Affiliations:** * NSW Department of Primary Industries, Yanco Agricultural Institute, Yanco NSW 2703, Australia; † NSW Department of Primary Industries, Wagga Wagga Agricultural Institute, Wagga Wagga NSW 2650, Australia; ‡ Charles Sturt University, Graham Centre, Wagga Wagga NSW 2678, Australia; § CSIRO Agriculture and Food, Canberra, ACT 2601, Australia; ** USDA-ARS Plant Science Research Unit and North Carolina State University Department of Crop and Soil Sciences, Raleigh, NC 27695-7620

**Keywords:** Genomic Selection, residual genetic variation, genotype-by-environment interaction, factor analytic model, relative accuracy, GenPred, Shared Data Resources

## Abstract

Abiotic stress tolerance traits are often complex and recalcitrant targets for conventional breeding improvement in many crop species. This study evaluated the potential of genomic selection to predict water-soluble carbohydrate concentration (WSCC), an important drought tolerance trait, in wheat under field conditions. A panel of 358 varieties and breeding lines constrained for maturity was evaluated under rainfed and irrigated treatments across two locations and two years. Whole-genome marker profiles and factor analytic mixed models were used to generate genomic estimated breeding values (GEBVs) for specific environments and environment groups. Additive genetic variance was smaller than residual genetic variance for WSCC, such that genotypic values were dominated by residual genetic effects rather than additive breeding values. As a result, GEBVs were not accurate predictors of genotypic values of the extant lines, but GEBVs should be reliable selection criteria to choose parents for intermating to produce new populations. The accuracy of GEBVs for untested lines was sufficient to increase predicted genetic gain from genomic selection per unit time compared to phenotypic selection if the breeding cycle is reduced by half by the use of GEBVs in off-season generations. Further, genomic prediction accuracy depended on having phenotypic data from environments with strong correlations with target production environments to build prediction models. By combining high-density marker genotypes, stress-managed field evaluations, and mixed models that model simultaneously covariances among genotypes and covariances of complex trait performance between pairs of environments, we were able to train models with good accuracy to facilitate genetic gain from genomic selection.

Abiotic stresses such as water deficit during the growing season are a major limitation to crop production worldwide (Fischer *et al.* 2014; Ray *et al.* 2015; Foulkes and Reynolds 2015). However, the incidence and severity, as well as the timing of water deficit can differ markedly between sites and years, contributing to the historically low rates of genetic gain for yield in water deficit environments compared to well-watered environments (Richards *et al.* 2010; Araus *et al.* 2002).

An alternative to selection for grain yield directly is to identify useful traits that confer physiological adaptation to water deficit conditions (Rebetzke *et al.* 2009; Bernier *et al.* 2008; Reynolds *et al.* 2016; Lopes *et al.* 2011). For example, selection for major genes for reduced height and photoperiod insensitivity has been used to alter plant architecture and provide drought escape potential in wheat (Passioura 1996; Bennett *et al.* 2012; Kamran *et al.* 2014). Water soluble carbohydrate (WSC) accumulation and remobilization can contribute to performance under water deficit as a source of assimilate for grain filling in many crop species (Slewinski 2012). Carbohydrate accumulation occurs when the crop synthesizes assimilate at a rate greater than needed by the various sinks (for example, developing florets, elongating shoots and roots). For wheat, the excess carbohydrate is stored mainly in the lower parts of stems and culms (Gebbing 2003), where the quantity of WSC can reach as much as 40% of total stem weight (Schnyder 1993). The main sink for remobilization of WSC is the developing grain (Schnyder 1993; Takahashi *et al.* 2001; van Herwaarden *et al.* 1998). Remobilized WSC can contribute as much as 30–50% of grain yield under terminal drought conditions, and around 10–20% under well-watered conditions (Bidinger *et al.* 1977; Schnyder 1993; Pheloung and Siddique 1991; Gebbing and Schnyder 1999).

Selection for increased WSC in cereal breeding programs has been advocated for some time (Blum 1998), and may be an alternative to direct selection for grain yield under water deficit conditions (Asseng and van Herwaarden 2003). Previous studies indicate higher broad-sense heritability for WSC concentration (WSCC) compared to grain yield, and more stable genotype rankings within a target population of environments (Ruuska *et al.* 2006; Dreccer *et al.* 2013; Piaskowski *et al.* 2016). Indirect selection for WSCC by breeders has already occurred in some environments. For example, Rebetzke *et al.* (2009) reviewed the trend for increasing WSCC with year of variety release observed for Western Australian and the International Maize and Wheat Improvement Center (CIMMYT) wheat breeding programs. Shearman *et al.* (2005) also reported that UK wheat cultivars showed increased WSCC with progressive year of release. However, WSCC is not predicted to be a useful indirect selection criterion for yield improvement in some cases (Ovenden *et al.* 2017); furthermore, phenotyping for abiotic stress characteristics such as WSCC is resource intensive and phenotypic selection may not always be feasible in a breeding program. The practical utility of WSCC for breeding appears to be context-dependent and requires empirical investigation for specific populations and environments.

New genomic approaches may be more efficient to select for abiotic stress traits than conventional phenotypic selection, as genomic selection can leverage the information obtained from difficult and expensive phenotyping (Lin *et al.* 2014; Cooper *et al.* 2014; Leplat *et al.* 2016), to enable more rapid and inexpensive selection for many loci that may be involved in the inheritance of these complex traits (Vivek *et al.* 2016; de los Campos *et al.* 2013). Comprehensive phenotyping is still an important part of the breeding process when genomic selection is applied, as it is essential to build accurate prediction models. However, early generation nursery screens can be replaced with genomic screens (Sallam *et al.* 2015), and expensive field trials can be augmented by genetic material ‘enriched’ through genomic selection (Heffner *et al.* 2009).

Genomic selection methods use marker-based measures of realized relatedness from whole-genome marker profiles to predict genomic estimated breeding values (GEBVs) of progeny (Habier *et al.* 2013; Meuwissen *et al.* 2001; de los Campos *et al.* 2013). For lines that have both genotype and phenotype data, GEBVs can be combined with the residual line effect estimates to produce Genomic Best Linear Unbiased Predictors (G-BLUPs) for the total genotypic value, hereafter referred to as the genotypic value (GV) as described in Oakey *et al.* (2016). As reviewed by Hill (2012); Nakaya and Isobe (2012) and de los Campos *et al.* (2013), a key difference between genomic and phenotypic selection is the potential reduction in breeding cycle time. Although genomic predictions of untested individuals are typically less accurate than well-replicated phenotypic evaluations, genomic selection may be more effective over time because of the opportunity to implement selection in additional generations per unit of time. If breeding cycles can be shortened, then relative accuracy can also decrease while still achieving greater genetic gain than phenotypic selection (Desta and Ortiz 2014).

Developing suitable genomic selection models for abiotic stress tolerance characteristics requires the consideration of complex genotype × environment interactions (hereafter G × E interactions) within and across a target population of environments because expression of these traits is often environmentally-dependent. Complex patterns of G × E interactions can be incorporated into genomic prediction models, and although G × E interactions will necessarily limit gains from selection for wide adaptation, models that incorporate G × E effects can help breeders select sets of lines optimally adapted to different subsets of environments. One such approach is to use parsimonious mixed models, such as factor analytic (FA) models, that attempt to capture most of the G × E signal with a reduced number of parameters compared to a full unstructured covariance model (Guo *et al.* 2013; Burgueño *et al.* 2012; Oakey *et al.* 2016).

The objective of this study was to evaluate the ability of genomic selection models to predict a complex physiological trait (WSCC) in both untested lines and in new environments using a mixed-model that accounts for variation in the pairwise correlations of performance in different pairs of environments.

## Materials and Methods

### Genotypes

The set of 358 lines used in this study was selected from a multi-site, multi-year irrigated winter cereals evaluation trial with a total of 1,314 genotypes. The genetic entries included both elite breeding lines and contemporary commercial varieties from Australian wheat breeding companies and CIMMYT representing a range of maturity types.

As WSC accumulation varies according to development stage (Ehdaie *et al.* 2008), this study aimed to assess genotypes as close as practicable to a common anthesis date. At the Yanco irrigated experiment in 2009, a total of 358 breeding lines and varieties out of the 990 grown in the experiment were selected based on common Zadoks’ development score (Zadoks *et al.* 1974) taken at approximately mid-anthesis. Lines selected were between Z49 (early head emergence) and Z56 (60% heading) which corresponds to a range of approximately 3-5 days difference in anthesis date in south-eastern Australia. For the second year of this study in 2010, the same breeding lines were selected for WSCC measurement except for 11 breeding lines that were excluded from the overall experiment in that year.

Lines were genotyped using the Illumina 9k Infinium iSelect beadchip array (Cavanagh *et al.* 2013) resulting in 4,883 polymorphic SNPs across the population (File S1). Missing values were imputed using Beagle (Browning and Browning 2009) implemented in the R package Synbreed (Wimmer *et al.* 2012). The resulting 4,162 SNP markers (excluding markers that were duplicated, monomorphic, and those with minor allele frequency of less than 5%) were used to compute a scaled identity by descent relationship matrix (K) after Endelman and Jannink (2012) (File S2). There was little evidence of population structure in the set of lines used in this study, with the first two eigenvectors of the K matrix accounting for approximately 15% of the observed variation in genomic relationships. A principal components plot of these eigenvectors showed no obvious clustering of lines (File S3).

### Experimental design

Experiments in this study were grown in south-eastern Australia at Coleambally and Yanco in 2009 and 2010. A split-plot design was used, in which the main-plot factor was irrigation treatment (irrigated or rainfed), and the 990 genotype entries (including the subset of genotypes for WSCC phenotyping) were the sub-plot factor. There were two replicates of each treatment at each site. The placement of genotypes within field experiment layouts was optimized with the spatial design package DiGGer (Coombes 2002). For the laboratory phase measuring WSCC using near-infrared spectroscopy (NIRS), an experimental design structured by day of measurement and NIRS instrument carousel and well was implemented to account for laboratory as well as field sources of experimental error. Samples from both field sites were pooled into one experimental design for each year, and the placement of genotypes within the laboratory experimental phase was also optimized with DiGGer (Coombes 2002), with partial replication of 20% of experiment field plots sampled (*i.e.*, a replication level of 1.20), following the methods in Cullis *et al.* (2006) and Smith *et al.* (2006).

Experiments were sown on a full profile of moisture, achieved by flood irrigating each site four to six weeks before sowing, so that the focus on water deficit conditions would be in the later stages of crop growth. Sowing dates were targeted for the first two weeks of May. Pre-sowing nitrogen was targeted to be approximately 120 kg N ha^-1^. Irrigated experiments were fertilized further to a total of approximately 300 kg N ha^-1^, consistent with the estimated nitrogen demand by the crops. Experiments were subject to a strict weed, pest and disease control regime to maximize yield potential. Irrigation scheduling for the irrigated treatments was intended to maintain soil water potentials above -100 kPa during the growing season, with irrigations commencing as soil water potential fell below -75 kPa.

### Water-soluble carbohydrate measurement

Tissue for WSCC analysis was sampled from a 50 cm long section of row (0.09 m^2^) in each plot when the irrigated treatments at each site were approximately 180° d post-anthesis, following the methods of Rebetzke *et al.* (2008). Approximately 5-10 stalks (including leaves, leaf sheaths and heads, but not senesced plant material) from each sample were ground to pass through a 2 mm sieve. Ground biomass samples were homogenized and subsampled for scanning by NIRS with a Bruker Multi-purpose Analyzer (Bruker Optik GmbH, Ettlingen, Germany) and OPUS software (version 5.1), and WSCC for the NIRS calibration samples (10% of the full set) was determined using the alkaline ferricyanide method (Piltz and Law 2007). For the 2009 experiment, the coefficient of determination for the calibration linear model was *r*^2^ = 0.92 and the root-mean-square error of cross-validation (RMSECV) was 15.4. For the 2010 experiment, the calibration linear model *r*^2^ = 0.92 and the RMSECV = 16.0. The WSCC phenotype data are provided in File S4.

### Statistical methods

A single-stage, multiplicative linear mixed model was used to analyze the multi-experiment data with the molecular marker data following the approaches of Beeck *et al.* (2010) and Oakey *et al.* (2016). The linear mixed model was:

$$y=X\tau +Z_{g}u_{a}+Z_{g}u_{\bar{a}}+Z_{u}u+\eta$$

where y is the n×1 data vector of the response variable across p experiments with N*_j_* plots per experiment j. Each combination of year (2009, 2010), site (YANA, COLE) and irrigation treatment (IRR, RFD) was treated as a separate experiment so that p=8. τ is a t×1 vector of fixed effects for the corresponding n×t design matrix (X), including experiment main and design-based effects. The term u is a random component with associated design matrix $Z_{u}\;$and contains experiment-specific terms used to capture extraneous variation (after Gilmour *et al.* 1997), including the blocking structure of the field (row, range, replicate and irrigation bay), and laboratory (day of measurement, instrument carousel and carousel well) phases of the design. The n×1 residual vector η was modeled within each year of the laboratory phase design. The m×1 vector of genetic line within environment effects g, with corresponding design matrix $Z_{g}$, is partitioned into a vector of additive line within environment effects $u_{a}$ and residual line within environment effects $u_{\bar{a}}$ such that $g=u_{a}+u_{\bar{a}}$, following the approach of Oakey *et al.* (2006). The difference between the ‘additive’ and ‘residual’ genetic effects is that the additive effects have a covariance structure proportional to the realized additive genetic relationship matrix, whereas the residual genetic effects are independent among lines. When we arrange the vectors of additive and residual genetic-within-environment effects ordered as *m* genotypes within each of *p* environments matrices, their covariance structures are:

$$var\left(u_{a}\right)=\;G_{ea}\;⊗\;K$$

$$var\left(u_{\bar{a}}\right)=\;G_{e\bar{a}}\;⊗\;I_{m}$$

where K is the m×m realized genomic relationship matrix estimated from the marker data described above and $\;I_{m}\;$ is an m×m identity matrix. For each variance model above, $G_{ea}$ and $G_{e\bar{a}}\;$are the p×p matrices of variances and covariances of additive and residual genetic effects across environments, respectively. Factor analytic models of different orders (different k) can be used to model the two genetic components (Smith *et al.* 2001). For a factor analytic model, these matrices are decomposed as

$$G_{ea}=\left(\Lambda_{ea}^{p\times k_{a}}\Lambda_{ea}^{’}\;+\;\psi_{ea}\right)\;$$

$$G_{e\bar{a}}=\left(\Lambda_{e\bar{a}}^{p\times k_{\bar{a}}}\Lambda_{e\bar{a}}^{’}\;+\;\psi_{e\bar{a}}\right).$$

Here, Λ is a p×k matrix of p environment loadings for k factors retained in the factor analytic model, and ψ is a diagonal matrix of the p environment specific variances.

A series of mixed models of increasing complexity of the $G_{ea}$ and $G_{e\bar{a}}$ terms were fitted to the data. First, we fitted diagonal covariance structures, in which the genetic variance was allowed to vary among experiments, but genetic effects were uncorrelated between experiments. Then a sequence of factor analytic models in which the covariances of genotype effects were allowed to vary within and among experiments were fitted to the data. Factor analytic models for each combination of k *=* 1 or 2 factors for each of the two genetic effects were used (Table 1). Selection of the final model was performed on the basis of Akaike’s Information Criterion (AIC); (Akaike 1974), and log likelihood ratio tests comparing the nested FA models (Stram and Lee 1994). All data were analyzed using the software package ASReml-R (Butler *et al.* 2009), in the R environment (R Development Core Team 2012).

**Table 1:** Factor analytic models fitted to the dataset for genomic prediction. Increasing order factor models were assessed using AIC and log likelihood ratio tests comparing nested models. The model with additive: FA2 and residual genetic: FA2 covariance structure shows a significant improvement in fit from both additive: FA1 / residual genetic: FA1 and additive: FA1 / residual genetic: FA2 models and is referred to as the final model. Higher order models were not possible to fit with the computing resources available

Covariance structure - Additive	Covariance structure - Residual genetic	REML Log Likelihood	AIC	Parameters	Full / reduced model parameters difference	Log likelihood ratio test model comparison:	Critical value	P value
DIAG	DIAG	4037.480	−8042.956	16	—	—	—	—
FA1	FA1	4405.638	−8747.276	32	16	to DIAG/DIAG	736.32	2.389×10^−146^
FA1	FA2	4465.921	−8853.842	39	7	to FA1/FA1	120.57	5.840×10^−23^
FA2	FA1	4453.568	−8829.136	39	7	to FA1/FA1	95.86	7.708×10^−18^
FA2	FA2	4473.418	−8854.836	46	7	to FA1/FA2	14.99	0.0361

Experiment-specific GVs incorporating both additive and residual genetic effects were obtained from the final model for each line following Beeck *et al.* (2010). The GV for line *i* at environment *j* was estimated from the random effect solutions in the final model as:

$$GV_{ij}={\hat{u}}_{aij}+{\hat{u}}_{\bar{a}ij}$$ (1)

These GVs were used later in cross-validation analyses as the best estimates of ‘true’ genotypic values at each experiment.

Additive genomic estimated breeding values (GEBVs) were also obtained using the same methods but based only on the additive genetic component of the model. The GEBV for line *i* at environment *j* was estimated from the random effect solutions in the final model as:

$$GEBV_{ij}={\hat{u}}_{aij}$$ (2)

Experiments were clustered based on the matrix of genetic correlations among experiments, using the agglomerative hierarchical clustering method given in Cullis *et al.* (2010).

Broad and narrow-sense heritability estimates were calculated from the final FA model (Table 1). Broad-sense heritability (H) for each trait at each experiment *j* was calculated following the generalized formula for unbalanced data in Cullis *et al.* (2006):

$$H_{j}=1-\frac{APPEV_{GVj}}{2\sigma_{aj}^{2}+2\sigma_{\bar{a}j^{2}}}$$

where $APPEV_{GTj}$ is the average pairwise prediction error variance of GVs at experiment *j* (the variance of pairwise GV differences), and $\sigma_{aj}^{2}$ and $\sigma_{\bar{a}j}^{2}$ are the additive and residual genetic variance components for experiment *j*, respectively. Narrow-sense heritability was calculated for each trait at each experiment using:

$$h_{j}^{2}=1-\frac{APPEV_{GEBVj}}{2\sigma_{aj}^{2}}$$

where $APPEV_{GEBVj}$ is the average variance of comparisons between GEBVs at experiment *j*, and $\sigma_{aj}^{2}$ is the additive genetic variance component at experiment *j*. The broad-sense heritabilities for total genotypic values of lines across all experiments and for genotypic values across experiments within each of the water deficit and well-watered environment clusters were also calculated using similar formulae. In these cases, however, we estimated the additive and residual genetic variances across experiments from the average of the additive and residual pairwise covariance estimates respectively between experiments for the experiments within each environment cluster (Zila *et al.* 2013; Isik *et al.* 2017).

### Cross validation of genomic estimated breeding values

Fivefold cross-validation (Ogut *et al.* 2015; Crossa *et al.* 2014; Burgueño *et al.* 2012; Lorenz *et al.* 2011) was used to measure accuracy of breeding value predictions across environments. Lines were randomly assigned to five subsets for a ‘fivefold’ cross validation scheme across separate experiments in the model. The final FA model (Table 1) was fitted to four of the five ∼80% subsets (‘training set’) to estimate model parameters and to predict GEBVs for the remaining subset (‘validation set’) (Equation 2). This procedure was repeated, holding out a different subset as the validation set each time. The entire process of allocating lines to folds, estimating model parameters, and predicting GEBVs was replicated ten times.

The GVs from the full model including all the data (Equation 1) were considered the best estimates of the true values of total genotypic value for each line-experiment combination. The correlation between the GVs within an experiment and the GEBVs for each validation set was estimated. We refer to these as within-experiment prediction accuracies. In addition, the ability of experiment-specific GEBVs to predict genotypic values at other experiments was estimated for each validation set as the correlation between the GEBVs for experiment *i* and the GVs from the full model at experiment *j*. We refer to these as across-experiment prediction accuracies.

Relative accuracy of phenotypic value prediction (RAPV) at experiment *j* measures the relative accuracy of GEBVs for untested lines compared to the best estimates of their total genetic value within each experiment using both genomic and phenotypic data:

$$RAPV_{j}=\frac{corr(GEBV_{ij},GV_{ij})}{\sqrt{H_{j}}}$$

In this case, the correlation value is divided by the square root of the broad-sense heritability estimate to estimate the accuracy of GEBVs relative to total genotypic value estimation with complete phenotypic data (Legarra *et al.* 2008). We also estimated relative accuracy of breeding value prediction at experiment *j* (RABV):

$$RABV_{j}=\frac{corr(GEBV_{ij},GV_{ij})}{\sqrt{h^{2}}}$$

The RABV compares the accuracy of GEBVs for untested lines relative to GEBVs based on complete data. The GEBVs are more informative to predict gain from selection after intermating selected lines to generate a new population of breeding lines.

### Data and reagent availability

The supplementary files contain the data used in this study. File S1 contains the SNP genotype information, File S2 contains the relationship matrix and File S3 contains a PCA plot of the first two eigenvectors of the relationship matrix. File S4 contains the phenotype dataset for WSCC. File S5 is script for the models used for genomic selection and cross-validation and File S6 contains the cross-validation sets. Supplemental material available at Figshare: https://doi.org/10.25387/g3.6143243.

## Results

### Model selection and genotype × environment interactions

**Figure 1:**
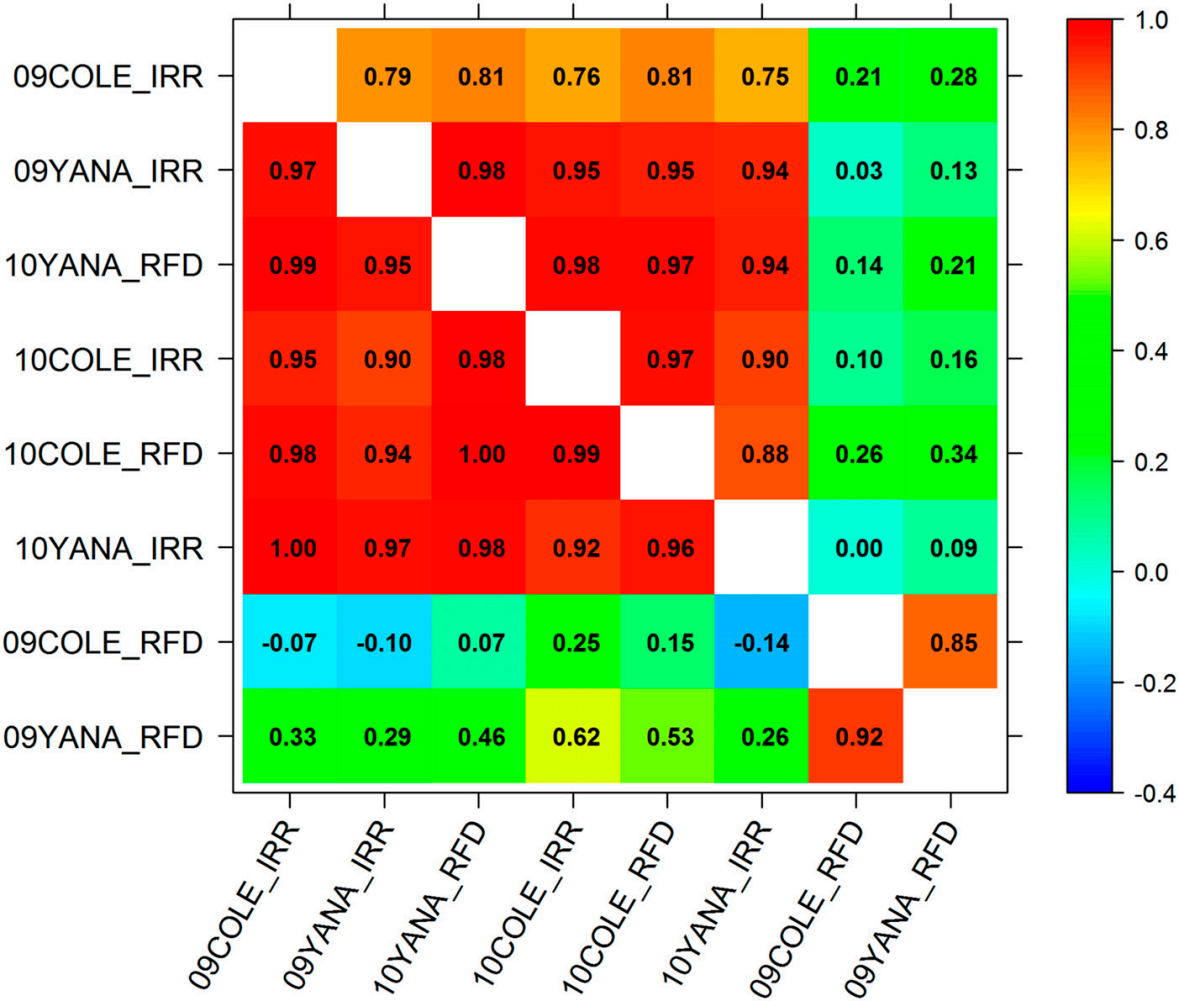
Correlations between total additive and residual genetic GV values in different experiments based on the full data set (above the diagonal) and correlations between additive GEBVs in different experiments, based on the full data set (below the diagonal). Experiment codes are given as year-site-irrigation treatment.

The 2-factor FA model for both additive and residual variance matrices was selected as the best model using the AIC (Table 1) and used for genomic prediction. The proportion of within-experiment total genotypic variance that was due to additive polygenic variance in this model ranged from 15 to 50% across experiments (Table 2). In the selected model, environments clustered into two distinct environmental groups based on the correlations between total genetic effects (GV) across experiments (Figure 1). The groups consisted of a well-watered environment cluster, including the 2009 irrigated experiments and all 2010 experiments, and a water deficit environment represented by the two 2009 rainfed experiments. This corresponded with environmental conditions encountered in this study. In 2009, both sites experienced below average rainfall, and warmer air temperatures, particularly during the later stages of grain-filling. Weather conditions throughout 2010 were cooler than average, with substantially above average rainfall during late spring (coinciding with the crop grain-filling period). Thus the non-irrigated experiments in 2010 did not suffer water deficit.

**Table 2:** Genetic variances, heritability, predictive ability and relative accuracy by experiment and environment cluster, with standard deviations given in parentheses. Experiment codes are given as year-site-irrigation treatment. The experiments 09YANA_RFD and 09COLE_RFD constitute the water deficit experiment cluster; all other experiments are included in the well-watered experiment cluster. The predictive ability of the GEBVs model at each experiment and environment cluster was divided by the broad-sense heritability to provide measures of accuracy relative to phenotypic selection (RAPV), and by the narrow-sense heritability to provide relative accuracy to total estimated breeding values (RABV)

Experiment or experiment cluster	Additive genetic variance	Residual geneticvariance	Proportion of genetic variance that is additive	Broad-sense heritability (H)	Narrow-sense heritability ($h^{2}$)	Predictive ability	Relative accuracy against H (RAPV)	Relative accuracy against $h^{2}$ (RABV)
All experiments	0.00824	0.01537	34.90%	0.778	0.363	0.480 (0.206)	0.544 (0.234)	0.797 (0.343)
Well-watered	0.01297	0.02299	36.07%	0.788	0.413	0.502 (0.192)	0.565 (0.220)	0.781 (0.304)
Water deficit	0.00914	0.03028	23.19%	0.810	0.181	0.455 (0.177)	0.506 (0.197)	1.070 (0.417)
09COLE_IRR	0.012713	0.043910	22.45%	0.853	0.345	0.503 (0.188)	0.545 (0.203)	0.857 (0.320)
09COLE_RFD	0.014623	0.031643	31.61%	0.760	0.242	0.471 (0.169)	0.540 (0.194)	0.958 (0.344)
09YANA_IRR	0.013688	0.023822	36.49%	0.791	0.427	0.535 (0.182)	0.602 (0.205)	0.819 (0.279)
09YANA_RFD	0.006774	0.039412	14.67%	0.891	0.260	0.445 (0.185)	0.471 (0.196)	0.873 (0.363)
10COLE_IRR	0.018323	0.021732	45.74%	0.722	0.392	0.474 (0.192)	0.558 (0.226)	0.757 (0.306)
10COLE_RFD	0.014472	0.014562	49.84%	0.777	0.552	0.466 (0.179)	0.529 (0.204)	0.627 (0.241)
10YANA_IRR	0.012508	0.042923	22.57%	0.862	0.388	0.520 (0.196)	0.560 (0.211)	0.835 (0.315)
10YANA_RFD	0.009967	0.020868	32.32%	0.806	0.400	0.481 (0.196)	0.536 (0.218)	0.760 (0.310)

Averaged across experiments in each environment cluster, the proportion of total genetic variance that was additive was 36% for the well-watered environment cluster, and 23% for the water deficit environment cluster (Table 2). Very similar patterns of relationships and clustering among environments were observed based on the correlations of additive-only genetic effects between environments (Figure 1), although the 09YANA_RFD experiment was less distinct from the well-watered cluster in this case than when the total genotypic correlations were considered. Correlations between the residual genetic effects of different environments also revealed a similar pattern, but with a slightly weaker correlation between 09COLE_IRR and the other well-watered environment experiments (Figure 1). The 09COLE_IRR experiment was the only experiment to be grown on raised beds with all other irrigated experiments utilizing a flat field layout.

### Cross-validation of genomic estimated breeding values

**Figure 2:**
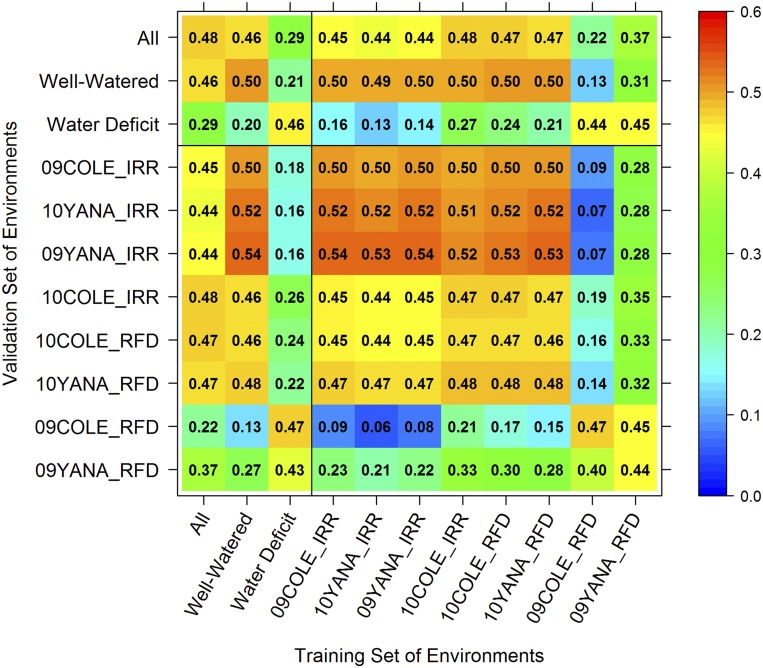
Predictive ability of GEBVs average across all experiments, averaged across experiments within each environment cluster (well-watered or water-deficit), or predicted for each specific experiment. The training set of environments is given by the X axis, and the validation set of environments is given by the Y axis. Experiment codes are given as year-site-irrigation treatment. Diagonal values represent ability of GEBVs within a given environment to predict GVs in the same environment. Off-diagonal values represent the ability of GEBVs in a given environment to predict GVs in a different environment.

The predictive ability (Sallam *et al.* 2015) of experiment-specific GEBVs was measured as the average correlation between experiment-specific GEBVs in the test sets and experiment-specific GVs estimated for the same lines when all trait data are used. Within-experiment prediction abilities ranged from r = 0.474 to 0.535 for the well-watered experiments and from r = 0.445 to r = 0.481 for the water deficit experiments (Table 2 and diagonal elements in Figure 2). The predictive ability across experiments within the well-watered environment cluster averaged *r* = 0.502, and across experiments within the water deficit cluster was *r* = 0.455 (Table 2 and diagonal elements of Figure 2). In contrast, GEBVs specific to an experiment within one cluster had much poorer predictive ability of GVs in the other cluster. We also estimated the ability of test set GEBVs specific to one experiment or cluster to predict GVs based on complete phenotype data in a different experiment or cluster (the off-diagonal elements of Figure 2). GEBVs for water-deficit experiments had only a weak correlation with the GVs in the well-watered environments (average *r* = 0.196). Similarly, GEBVs for the well-watered environments had an average correlation of r = 0.211 with GVs in the water deficit environments. Average GEBVs across all experiments also had low correlation with GVs within the water deficit environments (*r* = 0.287). Within each environment cluster, the GEBVs for individual experiments had moderate correlations with GVs at other experiments (*r* = 0.442 to *r* = 0.536 for the well-watered experiments, and *r* = 0.400 to *r* = 0.447 for the water deficit experiments).

The relative accuracy statistics were computed with both broad-sense and narrow-sense heritabilities as RAPV and RABV, respectively, so that a measure of accuracy relative to phenotypic selection can be compared to the accuracy relative to breeding value prediction (Table 2). As the broad-sense heritability estimates were much higher than narrow-sense, the relative accuracy statistics indicate that the efficiency of genotype value prediction was poor (RAPV ranged from 0.471 to 0.602 at individual experiments, with a mean of 0.544, Table 2), whereas the relative accuracy of breeding value prediction with marker information only compared to complete phenotype data were much higher (RABV ranged from 0.627 to 0.958 at individual experiments, with a mean of 0.797, Table 2). The RABV for the water deficit experiment cluster was 1.07, a surprising but valid result analogous to relative efficiency of selection on a correlated trait, which can be >1 relative to direct selection.

## Discussion

### Integration of genomic selection with genotype × environment effects

Our approach in this study combines the concept of a Target Population of Environments (TPE) in regard to selection (Comstock 1977; Cooper *et al.* 1997; Basford and Cooper 1998) with genomic prediction methods, and extends the work of Oakey *et al.* (2016) to a large field-based multi-environment trial. The TPE concept applies equally to genomic selection as phenotypic selection in the face of genotype-by-environment interactions. Our results show that predictive ability depends on the extent and nature of the genetic correlation between the training and the validation populations. Heslot *et al.* (2013) also demonstrated empirically in wheat that G × E patterns impact genomic selection in the same way that they impact phenotypic selection. Heslot *et al.* (2013) also observed that the main driver of prediction accuracy between environments were G × E effects and that genomic predictions are specific to the TPE they are predicted for – conclusions also supported by Lado *et al.* (2013) and Oakey *et al.* (2016), who also showed that multi-year models could give better prediction accuracy when environmental influence is large.

Genomic prediction models based on multi-environment trials may involve considerable complexity to allow heterogeneity of genetic correlations and genetic variances among environments, variable sources of extraneous non-genetic variation among environments, in addition to the high dimensionality of marker data. Researchers are faced with making choices about modeling greater complexity in the patterns of genetic correlations among environments *vs.* greater complexity in the genetic architecture modeled by marker data, based on tradeoffs between capturing more signal *vs.* overfitting and increasing computational demands as model complexity increases. A variety of modeling approaches have been proposed, reflecting different choices about which aspects of model complexity to emphasize. For example, Sallam *et al.* (2015) utilized an across-experiments model for TPE identified as having low G × E so the term in the model was minimized, whereas Crossa *et al.* (2010) modeled genetic effects within each environment separately. Lopez-Cruz *et al.* (2015) introduced a model with common G × E variance for all sets, but allowed variable marker effects through a Bayesian model. Heslot *et al.* (2014) and Jarquín *et al.* (2014) introduced models that accounted for marker interactions with specific climate variables, adding another layer of complexity to the modeling of G × E patterns.

Our focus in this study was to emphasize the modeling of complex patterns of heterogeneity in the genetic variation expressed within environments and the pairwise genetic correlations between environments, along with extraneous non-genetic effects. We chose a FA model (Cullis *et al.* 2010; Smith *et al.* 2001) that can capture such heterogeneity more parsimoniously than fully unstructured models. The patterns of genetic correlations between environments observed in training data sets and patterns of the accuracy of GEBVs for one site to predict genetic values at other sites in test sets from the FA model provide a way of characterizing target environments, still following the ideas on exploiting G × E to make genetic gains outlined by Byth (1981). The environment clusters in this study show that year effects are more important than location for WSCC (Figure 1), and this observation is borne out by other G × E studies with similar findings, particularly for traits where expression is significantly affected by seasonal conditions (Smith *et al.* 2015; Chenu *et al.* 2011; Milgate *et al.* 2015).

### Using factor analytic models for genomic predictions

Heslot *et al.* (2014) demonstrate a method to predict genotype performance in untested environments based on climatic variables. In contrast, the FA model approach helps breeders to understand the groupings of environments within the TPE based on their genetic correlations, and breeders can predict average performance in subsets of these environments. Both Burgueño *et al.* (2012) and Rutkoski *et al.* (2015) utilized similar FA models for genomic prediction, however this study shows that the power of an FA model lies in the ability to identify environment subsets based on the loadings and predict genotype performance more accurately within those TPE rather than the across-experiments average. Since we predicted values for untested lines within tested environments, our estimated prediction abilities are biased upward compared to prediction of untested lines within untested environments. Our results, which reflect upper bounds on prediction abilities across environments, demonstrate that GEBVs have reasonable accuracy only within a clearly defined cluster of environments, and have substantially worse prediction accuracy of performance in environments outside of the group of related environments for which they were predicted. The FA model permits prediction of genetic values for untested lines within each site based on the genomic relationships between the untested and tested lines and also based on the genetic correlations observed among the tested set of environments. The FA model can improve prediction accuracy within a single site over what is possible from a single-environment analysis by information sharing among environments with high genetic correlations (Guo *et al.* 2013; Cullis *et al.* 2010; Kelly *et al.* 2007). In this study, GEBVs averaged across all environments were less accurate for environment-specific prediction than GEBVs averaged across subsets of sites within the same environmental cluster (Figure 2).

A drawback to the FA model is that the emphasis on including more complexity in the modeling of extraneous genetic variation and the heterogeneity of genetic covariation among environments may limit the complexity of genetic architecture models that can be tested. The total genotypic value of each line was modeled as the sum of a polygenic additive effect and a residual genetic effect (after Oakey *et al.* 2016). Our results demonstrated that the residual genetic effects were more important than the additive effects in this case, which greatly limits the effectiveness of prediction of untested varieties, as those predictions depend only on the additive effects. The residual genetic effects were modeled as independent among varieties, such that they cannot contribute to prediction of untested varieties. Alternative strategies include explicitly modeling epistatic genomic relationships; we attempted this but could not achieve model convergence. Oakey *et al.* (2016) discuss the implications of including additional relationship matrices to account for a proportion of non-additive genetic effects, however they also note the difficulty of fitting several relationship matrices to a MET. Bayesian models could be fitted to the genomic relationships, to capture residual genetic relationships and variation among marker effects, but this approach would be even more computationally difficult; future research could focus on integrating heterogeneity of genetic covariances among sites into such models.

### Practical application of genomic selection for water soluble carbohydrate improvement

Breeders focusing on varietal development for target populations of environments that may experience abiotic stresses often seek to incorporate resistance to abiotic stresses, sometimes to contribute to grain yield *per se*, but also to select for stable grain yield performance in the presence of variable levels of abiotic stress. Thus, abiotic stress resistance traits can be a selection target in their own right, especially in situations where G × E variation is driven by abiotic stresses and resistance to these stresses can ensure grain yield stability across years. Genomic selection may be a good way to select for abiotic stress traits such as WSCC, especially as a substitute for resource-intensive phenotypic selection. Levels of WSCC increase and decrease with crop development and growing conditions, meaning that full expression of the phenotype is not easily captured, and both wet chemistry and NIRS methods for measuring water soluble carbohydrates are time consuming and expensive (Gebbing and Schnyder 1999; Ruuska *et al.* 2006).

We estimated that most of the genotypic variance for WSCC did not fit a polygenic additive polygenic model, a surprising result considering numerous results showing that wheat grain yield is often adequately described with an additive genetic model (Heslot *et al.* 2012; Pérez-Rodríguez *et al.* 2012; Burgueño *et al.* 2012). However, other studies of wheat yield across diverse environments indicate that residual genetic variation may explain substantial fraction of the total genetic variation (Cuevas *et al.* 2017). Because of the prevalence of residual genetic variance, the GEBVs for WSCC were not very accurate estimates of total genotypic value, regardless of the information used to compute them (markers, phenotype records, or both). In this study, residual genetic effects were modeled by fitting a separate independent random term for residual genetic effects in addition to the additive effects whose covariance is proportional to the additive realized relationship matrix (after Oakey *et al.* 2016). Total genotypic prediction, which includes additive and residual genetic effects, is optimal for identifying the best available lines in a population, but this requires phenotypic records on each line to be predicted. Potentially, predictions can be improved by explicit modeling of non-additive as well as additive genetic relationships, with either parametric models including dominance and epistasis (Muñoz *et al.* 2014; Su *et al.* 2012; Da *et al.* 2014) or non-linear kernel methods (Gianola and van Kaam 2008). These models would allow total genotypic values to be predicted on lines in the absence of any phenotypic records.

If the total genotypic values are not closely correlated with the true breeding values, they will not be the best predictions to use for parental selection to generate a new population from intermating, as dominance, epistatic, and most other non-additive effects that may contribute to the residual genetic effects do not contribute to long-term genetic gain over meiotic outcrossing generations (Hill *et al.* 2008; Holland 2001; Oakey *et al.* 2016; Cockerham 1983; Cockerham and Matzinger 1985). GEBVs, on the other hand, while they may not be optimal for predicting the best existing lines, should be better estimators of the utility of lines as parents of new breeding populations. To distinguish the different uses of GEBVs in a breeding program, we computed two relative accuracy statistics that refer to the use of GEBVs to identify optimal extant lines or to identify lines with better breeding values for use as parents for intermating to create a new breeding population. Compared to phenotypic selection the RAPV for the well-watered environment cluster was 57%, and 51% for the water deficit environment cluster, indicating our GEBVs are marginal at predicting phenotypic performance in these environments for new genotypes (Table 2), and that genomic selection is unlikely to be a substitute for phenotypic selection of the best performing lines. In contrast, when compared with true breeding values for the purpose of selecting new parents and maximizing genetic gain over time, the relative accuracy (RABV) was higher. The RABV measures can be coupled with the assumptions of Desta and Ortiz (2014); Heffner *et al.* (2010); and Lorenz *et al.* (2011) suggesting genomic selection is predicted to facilitate a reduction in the breeding cycle time of the average wheat breeding program of at least half due to the ability to select at earlier generations and also in off-season generations. Therefore, relative to selection using the true breeding values, and making the assumption of a decrease in the breeding cycle time of 50%, genetic gain per unit of time from genomic selection in this study is estimated to be 2×78%=158% for the well-watered environment cluster and 2×107%=214% for the water deficit environment cluster. This indicates that genomic selection methods can increase the rate of genetic gain for WSCC. The challenge in practical terms, as noted by Cooper *et al.* (2014), may be in implementing the enabling technologies needed to make genomic selection work and readily integrated into a commercial breeding program. Finally, if the ultimate goal of selection is to improve yield performance within or across abiotic stress environments, the genotypic and additive genetic correlations of WSCC with yield and other agronomic traits must be considered. Ovenden *et al.* (2017) previously demonstrated significant but relatively low genotypic correlations between WSCC and yield in this germplasm sample, suggesting that its utility as an indirect selection criterion needs to be assessed on a case by case basis.

### Conclusions

This study provides empirical evidence that genomic selection methods could improve the rate of genetic gain for carbohydrate accumulation, provided that the TPE are carefully characterized and understood, and predictions are restricted to environment subsets of interest.

With additive variance being low in the models under study, the importance of relative accuracy for different genomic selection strategies becomes apparent. Compared to phenotypic selection, the relative accuracy of the GEBVs modeled here is low, however the GEBVs more accurately model the true breeding values. Therefore, genomic selection could well result in higher genetic gain per unit of time through the ability to better select parents for intermating, and genomic selection may be a useful tool for making genetic gains in complex abiotic stress characteristics.
